# Conservation implications of using an imitation carnivore to assess rarely used refuges as critical habitat features in an alpine ungulate

**DOI:** 10.7717/peerj.9296

**Published:** 2020-06-12

**Authors:** Wesley Sarmento, Joel Berger

**Affiliations:** 1 Wildlife Biology Program, University of Montana, Missoula, MT, USA; 2 Montana Fish, Wildlife and Parks, Conrad, MT, USA; 3 Colorado State University, Fort Collins, CO, USA; 4 Wildlife Conservation Society, Bronx, NY, USA

**Keywords:** Anti-predator, Predation risk, Mountain goat, Predator-prey, Resource selection, Oreamnos americanus, Fear, Predation refugia, Security habitat optimization, Harvest

## Abstract

Understanding relationships between animals and their habitat is a central goal in ecology with important implications for conservation. Misidentified habitat requirements can have serious repercussions because land protection or reintroductions might occur in less than optimal habitat. Studies of resource selection have greatly facilitated an understanding of ecological relationships but can be improved when vital yet infrequently utilized habitat features are more fully described. A critical element for many prey species is escape terrain or some other form of refuge to avoid predation. Mountain goats (*Oreamnos americanus*) are well known for their use of cliffs to avoid predation, but a survey of the literature revealed at least twelve different approximations of goat escape terrain, ranging from > 25° to > 50° slopes. Here, we seek to (1) enhance estimates of mountain goat escape terrain and antipredator behavior, and (2) highlight the limitations of the assumption that the time an animal spends in an area is proportional to importance. To improve estimates of goat escape terrain, we conducted field work across two years (2014–15) in Glacier National Park, Montana USA and manipulated apparent predation risk by exposing mountain goats to a threatening simulated grizzly bear (*Ursus arctos*) treatment and a non-threatening ungulate (control) treatment. Mountain goats moved in response to the simulated bear but not in response to the simulated ungulate, with shorter latencies to move for subjects in larger groups and at shorter distances to the simulated threat. Through a used-unused resource selection function we tested 22 landscape variables to describe the use of escape terrain. Proximity to slopes greater than 60° best explained the locations to which mountain goats fled after exposure to the simulated bear, and the average slope of these escape locations was 56.5° (±14.1 S.D.). Our results suggest that mountain goat escape terrain be considered at slopes of 60° as a minimum because our simulated threat did not include pursuit of goats and, thus, slopes of 60° are likely underestimates of actual escape terrain. Additionally, because direct interactions between carnivores and goats seldom occur, serious escape terrain is infrequently used. Past estimates of escape may have miscalculated the slopes which goats select for in response to predation risk. Based on experimental approaches in the wild, we suggest that anti-predator behavior should be included in studies of resource selection when the goal is to consider habitat as a predictor for conservation success. Finally, we discuss evidence suggesting a past mountain goat introduction failed due to lack of adequate escape terrain and subsequent recolonization of a predator.

## Introduction

Effective *in situ* conservation depends on correct identification of habitat requirements because such ecological metrics are fundamental to planning introductions and land protection (Mitchell et al., 2012). To identify habitat requirements for particular species, most researchers rely on some form of resource selection or patch occupancy analysis (Manly et al., 2002). Resource selection studies are extraordinarily useful, but often contingent on a critical assumption—that the time an animal spends with a resource relative to availability is proportional to importance (Beyer et al., 2010). This assumption may be violated during a trade-off, such as habitat selection that is density dependent or influenced by interactions with predators (Mitchell et al., 2012). Encounters between prey and predator are generally rare. Consequently, such events of biological and evolutionary importance may often go undetected. Interactions with predators also unduly impact the assumption that the time spent with a resource is proportional to importance, because fitness consequences are associated with reliance on refuge habitats (Sih, 1987). Such locales can be broadly defined to include any area that decreases predation risk. The ecological importance of recognizing how prey species select refuge habitat has conservation implications, especially in those cases where reintroduction or range alterations contribute to population persistence.

Species and sexes differ in their need, form, and use of refuge habitats (Stankowich & Blumstein, 2005; Stankowich, 2008). Among large mammals, refuge habitat has received attention due to economic and societal interest. For example, vegetative cover was once considered the most important habitat feature for elk *(Cervus canadensis*) to escape human hunters, but it is now clear that limited human access is more central than hiding cover, a result that would not have been detectable in the absence of powerful resource selection models (Proffitt et al., 2013). In other ungulates, such as Dall and bighorn sheep (*Ovis dalli, O. canadensis),* refuge habitat consists of topographic features—frequently referred to as escape terrain, which specifically has been defined as “rockland with steep slopes on which individuals can outrun predators” (Rachlow & Bowyer, 1998; Hoglander et al., 2015). Escape habitat has often been classified vaguely, without actual quantification, which has led to inconsistencies within species. For example, for Tibetan argali (*Ovis ammons*), escape terrain has been considered as both “cliffs” and as “gentle slopes in open landscape,” in different studies being carried out in similar systems (Namgail, Fox & Bhatnagar, 2004; Singh et al., 2010). A better understanding of prey animal refuge habitats is of both conceptual and pragmatic value because it enables insights about interspecific ecological relationships and associated habitat as well as distinguishing between sites where conservation may succeed or fail, particularly where population restoration may be a goal.

Mountain goats (*Oreamnos americanus*) are a rupicaprid with an ancestry that presumably was tied to cliff-like escape terrain (Festa-Bianchet & Côté, 2008). In areas with higher predation risk, goats trade off forage for safety by staying closer to cliffs (Hamel & Côté, 2007). Escape terrain may be important beyond direct interactions with predators since goats select for areas closer to cliffs during vulnerable activities such as sleeping (Sarmento & Berger, 2017). Furthermore, ledges surrounded by vertical cliffs are important sites where females give birth and rear young during the first days to reduce predation risk (Chadwick, 2002). Flatter ledges and gentler slopes provide mountain goats with most of their forage because these places retain soil and water for plant growth.

Slope is perhaps the single best feature to characterize escape terrain because rock landcover can occur at varying angles and predators can readily move across rocks on gentle slopes. The wide availably of rock landcover would not accurately describe the steep slopes that goats need to escape carnivores. Descriptions of goat escape terrain vary widely from slopes ≥ 25° to ≥ 50°, and little is known of the extent to which goats preferentially select steeper areas during interactions with predators (Table 1). Certainly, availability and context specificity can help explain part of the variability in prior estimates of escape terrain for mountain goats, but there must be some sort of minimum slope necessary to facilitate successful avoidance of predation. Further, different methods of habitat estimation may contribute to inconsistency in definitions. More recent analyses have employed resource selection functions (RSFs) where spatial importance is proportional to the amount of use. RSFs have vulnerabilities, especially when habitat features may be under-represented in their use (Ciarniello et al., 2007). Because goat-predator interactions rarely occur, crucial escape terrain is accessed at disproportionately low frequency compared to areas regularly used for foraging. Consequently, relatively gentler slopes have been designated as optimal escape terrain because such topography is used most frequently. DeVoe et al. (2015), for instance, tested various mountain goat escape terrain slope angles using occupancy models at a coarse scale with a 100-m cell size which may have under-represented critical, but spatially scarce, habitat features. These past studies have sought to define and quantify escape terrain without actual observation of predator interactions; consequently, these estimates are often educated approximations or a proportional averaging of slopes where goats most often occurred relative to availability. Thus, a clear knowledge gap exists concerning the topographic characteristics of habitat used by goats during interactions with predators.

**Table 1:** Summary of definitions of mountain goat escape terrain in scientific publications, professional reports, and graduate theses. All native carnivores were present at each location, except in Colorado and Washington where grizzly bears and wolves had been extirpated.

**Definition**	**Location**	**Citation**
multiple	Wyoming	Lowrey et al. (2017)
multiple	Montana	DeVoe et al. (2015)
cliff	Alberta	St-Louis et al. (2013)
cliff	Alberta	Hamel & Côté (2007)
>25° slope	Montana	Varley (1996)
> 33° slope	Colorado	Gross et al. (2002)
>35° slope	Washington	Wells et al. (2014)
> 40° slope	Alberta	Richard et al. (2014)
>40° slope	Alaska	Shafer et al. (2012)
> 40° slope	British Columbia	Poole et al (2009)
>45° slope	British Columbia	Poole and Heard (2003)
>50° slope	Alaska	White and Gregovich (2017)
>50° slope	British Columbia	Taylor et al (2004)
>50° slope	Alaska	Smith (1994)
>50° slope	Alaska	Fox et al (1989)
45–60° slope	British Columbia	Lele et al. 2013

**Notes.**

a DeVoe et al., 2015 estimated a 37° slope as the optimal for goat occupancy.

We attempted to deepen insights about escape terrain habitat selection in mountain goats by examining responses to grizzly bears (*Ursus arctos*), a key predator throughout their native range (Côté & Beaudoin, 1997; Festa-Bianchet & Côté, 2008). Because interactions between mountain goats and grizzly bears are rare and difficult to observe, we experimentally simulated encounters by using an imitation grizzly bear (bear simulation). In addition, we contrasted goat responses to a simulated bighorn sheep, as a control to ensure goats were responding to predation risk and not novelty. Our goals were to (1) enhance estimates of mountain goat escape terrain and antipredator behavior, and (2) highlight limitations of the assumption that the time an animal spends in an area is proportional to importance. We combine our simulated bear experiment with a used-unused conditional logistic regression model to evaluate components of landscape variables associated with escape terrain. Further, we contextualize the generality of our results derived from field experiments through comparison with habitats documented in actual predator-goat encounters in the wild.

## Materials & Methods

### Study site

Glacier National Park (48.6967° N, 113.7183° W), Montana, USA is situated in the northern Rocky Mountains along the Canadian border. The Continental Divide splits the region into two climate zones; Western slopes are dominated by inland Pacific maritime weather whereas Eastern slopes experience Arctic continental weather systems. Precipitation is highly variable depending on location relative to the divide and elevation which ranges from 960 m to 3,190 m. The park contains a full suite of native carnivores including wolves (*Canis lupus*), mountain lions (*Puma concolor*), grizzly bears, black bears (*U. americanus*), wolverines (*Gulo gulo*), golden eagles (*Aquila chrysaetos*), and coyotes (*C. latrans*). In 2009 the 4,100 km^2^ park was estimated to contain 1,885–3,269 mountain goats (Belt & Krausman, 2012). We define habitat as the suite of resources and conditions, both biotic and abiotic, necessary for survival and reproduction (Sinclair, Fryxell & Caughley, 2006). Our research conformed to an institutional animal care and use committee permit (Permit # 017-15) from the University of Montana and all methods were approved by Glacier National Park (GLAC–2013–SCI–007).

### Experiment and rationale

We examined the extent to which mountain goats perceive and respond to predation risk during interactions with familiar dangerous and neutral species. We assumed that flight would reveal critical aspects of refuge habitat from a predator and explored the behavioral consequences of staged interactions in a fine-grained fashion. Specifically, we manipulated the apparent presence of predators by presenting visual simulations of different risk: (1) grizzly bear (potential danger), and (2) a familiar ungulate, bighorn sheep (low risk), as a control. We expected goats to respond weakly to the familiar ungulate treatment, but more strongly to a carnivore treatment since grizzly bears kill and consume goats (Côté & Beaudoin, 1997). Our reliance on a non-risk control was critical to test the hypothesis that goats discriminate between the risk putatively posed by our models. If goats fled the ungulate treatment at a similar frequency and intensity as the bear treatment, then the obvious inference would be that novelty—perhaps because of non-realistic models or an unlikely lack of recognition of species per se—rather than treatment type governed responsiveness. We conducted our experiments in June–September of 2014–15.

In 2014, the familiar ungulate treatment consisted of a researcher using a bighorn sheep head constructed from foam (Delta McKenzie Targets, Inc.; Salt Lake City, Utah), a beige shirt/pants, and foam to resemble front legs. The 2014 grizzly bear treatment was a styrofoam bear head and furred fabric cape. Treatments were reconfigured in 2015 to decrease weight for improved transport and accessibility to remote sites more than 15-km into the backcountry. Goats in these realms away from roads are unfamiliar with people, and often stare or flee upon encounters with humans. In 2015, the familiar ungulate treatment was a researcher dressed in all beige with a deer (*Odocoileus virginianus*) hunting decoy repainted in bighorn sheep coloration (Montana Decoy Company; Hummelstown, Pennsylvania) and foam front legs. The 2015 grizzly bear treatment was a researcher with a large dark brown coat, hat and pants combined with a bear mask (Rubies Costume Company; New York, New York). In a previous study testing broad differences in anti-predator behavior between habituated and wild goats we found that the reconfiguration of the stimuli did not have a significant effect (Sarmento & Berger, 2017).

To start a trial we located an accessible group of goats not already on escpae terrain at one of five salt licks spread sufficiently distant from each other such that we were confident that we did not repeat trials on the same individuals. Our experimental exposures were not presented to goats already on escape terrian (>60° slopes). We hiked undetected to within 200 m of the group and remained hidden in vegetation. We randomly selected a focal individual by observing the time and then using the minute’s count as a random number. Then we counted off individuals from left to right until we reached that minute’s number –e.g., if we began an observation at 11:23, we would count off—cycling back through a group, if needed—until we reached the twenty third, which would be our focal goat. If a focal goat was out-of-sight at that moment, then we either waited for the individual to become visible or we selected the next goat to the right.

We used a before-during-after impact research design where three 180-s focal samples were conducted—one prior, one during, and one 12 min after treatments ended. Based on a pilot season, we chose 12 min after treatment exposure because goats frequently became difficult to observe in the cliffs as more time elapsed. First, both the data recorder and technician remained hidden in vegetation while pre-treatment data were collected. Then, the data recorder remained hidden in vegetation while a field technician left the vegetation to conduct the treatment. All treatments were presented to the focal subject during daylight via a lateral view of the simulated bear or ungulate. We never directly approached goats with the stimuli. After the 180-s treatment the technician returned to the vegetation to hide. Both biologists then remained hidden and collected the final focal sample 12 min after the treatment ended.

In 2014, we randomized order of the treatments to control for potential sequence effects. If the individual under observation remained in the location after the initial treatment, we then performed the alternative treatment presentation once the exposed individual (1) re-initiated its pre-disturbance behavior, and (2) 30 min had elapsed. We did not repeat the same treatment within the same group on the same day. Because escape reactions were consistent within treatments in 2014, we switched to presenting treatments in increasing order of apparent threat (ungulate, then bear) in 2015. We opted for this ordering in 2015 because goats were always more likely to run from the simulated bear than the ungulate. All other protocols and treatment criteria remained the same between years. Goats frequently distanced themselves from us after exposure to the bear stimulus resulting in fewer ungulate treatments when the bear was presented first. In our prior work in which we assessed broad differences in anti-predator behavior between habituated and wild goats we found that neither year nor the sequencing of treatments had a detectable impact on responses (Sarmento & Berger, 2017).

The 180-s focal samples included the following explanatory variables: location, sex/age class of focal individual, group size and composition, distance to nearest neighbor, distance between treatment and the subject, distance between observer and subject, wind, and treatment type. Goat location was identified using a rangefinder and topographic map within a Garmin E-Trex Vista Global Positioning System with an accuracy of 3m. The nearest neighbor was the distance from the focal goat to the closest adult individual and was estimated as the number of adult goat lengths (about 2m) between individuals. Other distances were estimated using a rangefinder. Wind speed was recorded with a Kestrel 2000, and treatments were always presented downwind from subjects.

Response variables were behavior, movement rates, latency to response, and escape location 12 min after stimulus ended. We recorded behaviors as time spent engaging in each activity during the 180-s focal samples that occurred during the treatments. Operationally, we classified feeding when a goat was biting or smelling vegetation with its head below the shoulders. Vigilance was defined when an individual raised its head at, or above, the shoulders and was stationary. Movement behavior occurred when an individual stepped and held its head at or above the shoulders. Additionally, latency to response was the time between stimulus detection by a focal goat and flight, which was recorded during treatments. Detection was judged to have occurred when the focal goat became vigilant towards the stimulus. Similarly, time to group clustering was defined as elapsed time between detection of the treatment stimulus and family groups of goats moving to within five meters of each other and was also recorded during treatments. Various time counts were recorded using three stopwatches.

### Antipredator behavior analyses

We assessed the possibility of differences in behavioral responses to treatments by comparing the time allocated to each behavioral category. We tested differences in movement between baseline (pre-treatment) and treatment values using Welch’s two-sample t-tests which corrects for unequal variance. Alpha level was set a priori at 0.05. Latency to response (e.g., time before flight after detection) was tested using univariate linear models. To understand which covariates best explained goat latency to response, we tested both untransformed and normalized data. Models explaining variations in latency to response were ranked using the small sample size corrected Akaike Information Criterion (AICc) scoring (Burnham, Anderson & Huyvaert, 2011). Data were analyzed in the statistical program R (R Development Core Team, 2015), and no warning messages were produced, although diagnostic plots exposed three data points as outliers. We retained these three data outliers because they did not influence the univariate AICc rankings nor coefficient estimates by more than 20%.

### Escape terrain selection analyses

To gauge the escape terrain used by goats, we used proxy measures of the topography to which goats fled after our bear stimulus treatments. We obtained remotely sensed landscape explanatory variables in a geographic information system (GIS) using ArcGIS 10.2 (ESRI, Redlands, California). Using an Advanced Spaceborne Thermal Emission and Reflection Radiometer (ASTER) 10-m digital elevation model (DEM) raster layer (earthexplorer.usgs.gov, accessed 10 March 2016), we produced other terrain variables at the same resolution: slope and distance to slopes 30, 40, 50, 60, and 70 degrees. Then, with the ArcMap focal statistics and raster calculator tools we created slope variability maps: a 100-m resolution slope variability (SV100 = Slope_max_ –Slope_min_) map, and another at 10-m resolution (Ruszkiczay-Rudiger et al., 2009). Additionally, we created a 10-m slope variability map based on the DeVoe et al. (2015) designation (standard deviation of slope squared). We also created a ruggedness map via the vector ruggedness tool (Sappington, Longshore & Thompson, 2007). Next, we included a map of terrain curvature (Walker et al., 2007). Finally, we included a Moderate Resolution Imaging Spectroradiometer 17 class vegetation map to test for selection of landcover types during escape (http://www.modis.gsfc.nasa.gov, accessed 10 March 2016).

We examined habitat attributes of locations that goats used to escape our bear simulation treatments, which we defined as the location of the focal subject 12 min after exposure to stimulus. We used univariate conditional logistic regression models with the clogit function in the survival package in R to determine which habitat features were selected for during escape (Therneau & Grambsch, 2000). At each escape locale, we randomly selected five additional unused points based on a distribution of distances fled which we compiled using Geospatial Modeling Environment (GME) (Beyer, 2012). Conditional models contrasted actual locations of flight to the five random sites. Used-unused points were paired with associated landscape covariates using GME. For variables that were not normally distributed, we tested transformed versions. AICc was used to rank models. The landcover covariate model did not converge due to lack of differences between escape and available locations and thus could not be included in analyses; both used and unused locations were rock cover. We tested the predictive accuracy of our model using k-fold cross-validation with 10 folds using the cvbinary function in the DAAG package (Boyce et al., 2002). There is no accepted k-folds threshold that represents a good model fit; however, a value closer to 1.0 suggests a valid model. Finally, we estimated the area under the receiver operator characteristics curve (AUC) to estimate predictive ability of our model—where an AUC value of 1.0 represents a perfect prediction (Swets, 1988).

## Results

### Antipredator behavior

Sample sizes were as follows: pre-stimuli baseline (67), simulated ungulate (31), and simulated bear (37). We obtained more simulated bear samples because once exposed to the simulated bear, focal goats disappeared among cliffs, which prevented us from conducting an ungulate treatment. In one instance we were not able to obtain a pre-stimulus sample because a goat was walking closer and about to detect the biologists hidden in the trees. Our simulated trials included the following age and sex groups: (1) baseline - 28 females with young, 9 females without young, 28 adult males, and 2 juveniles, and (2) ungulate treatments - 10 adult females with young, 5 adult females without young, and 16 adult males. Bear treatment metrics were collected on 18 adult females with young, 4 adult females without young, 13 males, and 2 juveniles.

**Figure 1:**
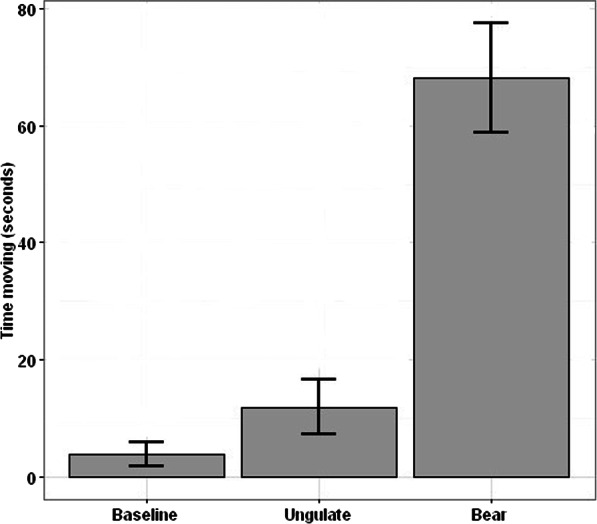
Mountain goat movement during experimental presentations. Mean (with 95% CI) number of seconds that focal mountain goats were moving within 180-s observations pre-treatment (baseline) and after exposure to the control (ungulate) and experimental (bear) treatments. Samples sizes are: baseline* = 67, ungulate treatment = 31, bear treatment = 37, in Glacier National Park, Montana, USA during June, July, and August of 2014–15. *baseline data were unavailable in one instance.

Group sizes were similar between baseline and post-treatment exposures: baseline 4.84 goats (S.D. = 4.3, Range 1–21), ungulate 4.74 (S.D. = 4.9, Range 1–21), and bear 4.54 goats (S.D. = 3.2, Range 1–14). Baseline movement was 3.84 s (2.13% of sample time) on average (S.D. = 18.5, Range 0–127). Movement 12 min after the ungulate treatment was 11.82 s (6.57%) on average (S.D. = 25.1, Range = 0–106) and did not differ from baseline (*t* =  − 1.54, *df* = 38, *p* = 0.131). Twelve minutes after the bear treatment mountain goat movement was 68.14 s (37.86%) on average (S.D. 57.3, Range = 0–180), and was increased from baseline by 64.3-s (Fig. 1; *t* =  − 6.08, *df* = 34, *p* < 0.001).

The top four models explaining latency to respond to the bear treatment (time between detection and flight) carried 98% of the AICc weights (Table 2). Latency to respond to the bear treatment was most influenced by group size, with goats in larger groups responding more quickly to the simulated bear (ß= 0.14, S.E. = 0.16, *Z* = 0.93, *P* = 0.35; Table S2) and was positively correlated with the distance to the bear stimulus, where goats fled more quickly when closer to the stimulus (ß= − 0.01, S.E. = 0.003, *Z* =  − 2.34, *P* = 0.02; Table  S1). Distance to the simulated bear ranged from 20 m to 413 m (mean = 89.2 m, S.D. = 115.6 m). Because the mountain goats did not flee from the ungulate treatment, there was no latency to response.

**Table 2:** Mountain goat latency to response after exposure to a bear simulation. Summary of univariate variables explaining mountain goat latency to response (time until flight after detection of treatment) after exposure to a bear treatment (values reported are based on linear models).

**Model**	**K**	**AICc**	**ΔAICc**	**AICcWt**	**LL**
Group size	3	375.97	0.00	0.41	−184.51
Log group size	3	376.19	0.22	0.37	−184.61
Distance to treatment	3	377.81	1.84	0.16	−185.44
Log distance to treatment	3	381.07	5.10	0.03	−187.07
Distance to observer	3	383.05	7.08	0.01	−188.06
Log distance to observer	3	385.82	9.85	0.00	−189.45
Log distance to >60° slopes	3	387.79	11.82	0.00	−190.44
Wind	3	388.38	12.41	0.00	−190.73
Log wind	3	388.39	12.42	0.00	−190.74
Distance to >60° slopes	3	388.44	12.47	0.00	−190.76
Temperature	3	388.52	12.54	0.00	−190.80
Log temperature	3	388.52	12.54	0.00	−190.80
Initial behavior	6	389.57	13.59	0.00	−186.96
Sex/age	5	392.02	16.05	0.00	−189.76

**Table 3:** Influence of landscape features explaining locations to which mountain goats had fled after exposure to a bear stimuus. Summary of univariate influences of landscape features explaining locations to which mountain goats fled after exposure to a bear treatment. Results are from used-unused conditional logistic regression models. SD, standard deviation.

**Model**	**AICc**	**Δ AICc**	**Weight**	**Log Likelihood**
Distance to slopes >60°	25.70	0.00	0.62	−11.84
Log distance to slopes >60°	27.84	2.14	0.21	−12.91
Distance to slopes >50°	28.83	3.13	0.13	−13.41
Slope	32.40	6.70	0.02	−15.19
Log slope	33.17	7.47	0.01	−15.57
Log distance to slopes >40°	36.74	11.04	0.00	−17.36
Log distance to slopes >50°	37.50	11.80	0.00	−17.74
Log distance to slopes >70°	44.92	19.21	0.00	−21.45
Distance to slopes >40°	48.10	22.40	0.00	−23.04
Log SD (slope)^2^ 10 m	52.71	27.01	0.00	−25.34
SD (slope)^2^ 10 m	59.21	33.51	0.00	−28.59
Log slope variability 10 m	59.44	33.74	0.00	−28.71
Elevation	61.45	35.75	0.00	−29.71
Slope variability^a^ 10 m	62.20	36.49	0.00	−30.09
Log distance to slopes >30°	72.14	46.44	0.00	−35.06
Distance to slopes >30°	74.83	49.13	0.00	−36.40
Distance to slopes >70°	75.47	49.77	0.00	−36.72
Slope variability^a^ 100-m	104.07	78.36	0.00	−51.02
Log slope variability 100-m	104.09	78.38	0.00	−51.03
Curvature	104.50	78.80	0.00	−51.24
Ruggedness	105.94	80.23	0.00	−51.96

**Notes.**

a Slope variability is the slope max-slope minimum. Slope variability was tested at both 10 m and 100 m resolution

Group clustering did not occur during any of the bear or ungulate treatments. Nearest neighbor distances did not differ significantly between before (mean = 3.21 m, S.D. = 4.6) and after (mean = 2.45-m, S.D. = 4.1) the bear treatment (*t* = 0.76, *df* = 50, *p* = 0.45). There were no differences between years in response to treatments (as previously reported, Sarmento & Berger, 2017).

### Escape terrain selection

**Figure 2:**
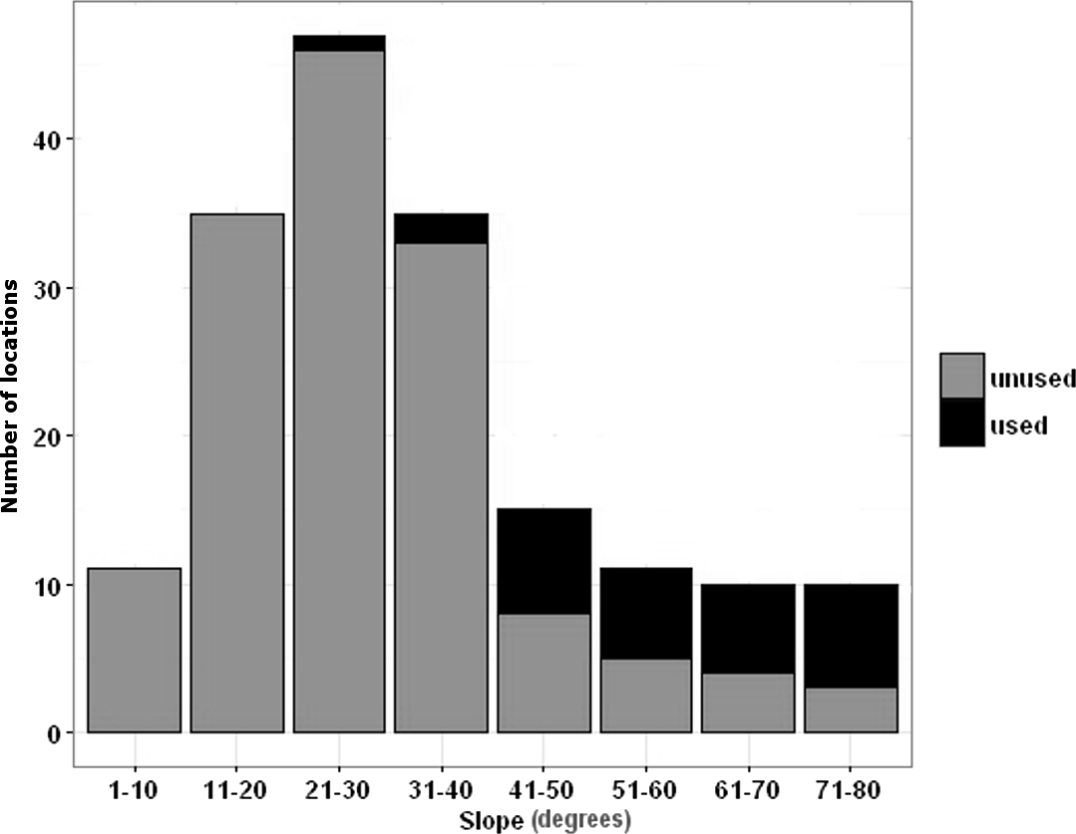
Slopes that mountain goats used or did not use after grizzly bear experiments. Frequency distribution of available slope angles at locations mountain goats used or did not use 12 minutes after grizzly bear stimulus ended in Glacier National Park, Montana, USA during June, July, and August of 2014–15. Sample sizes are one used and five unused locations for each of 37 trials.

**Figure 3:**
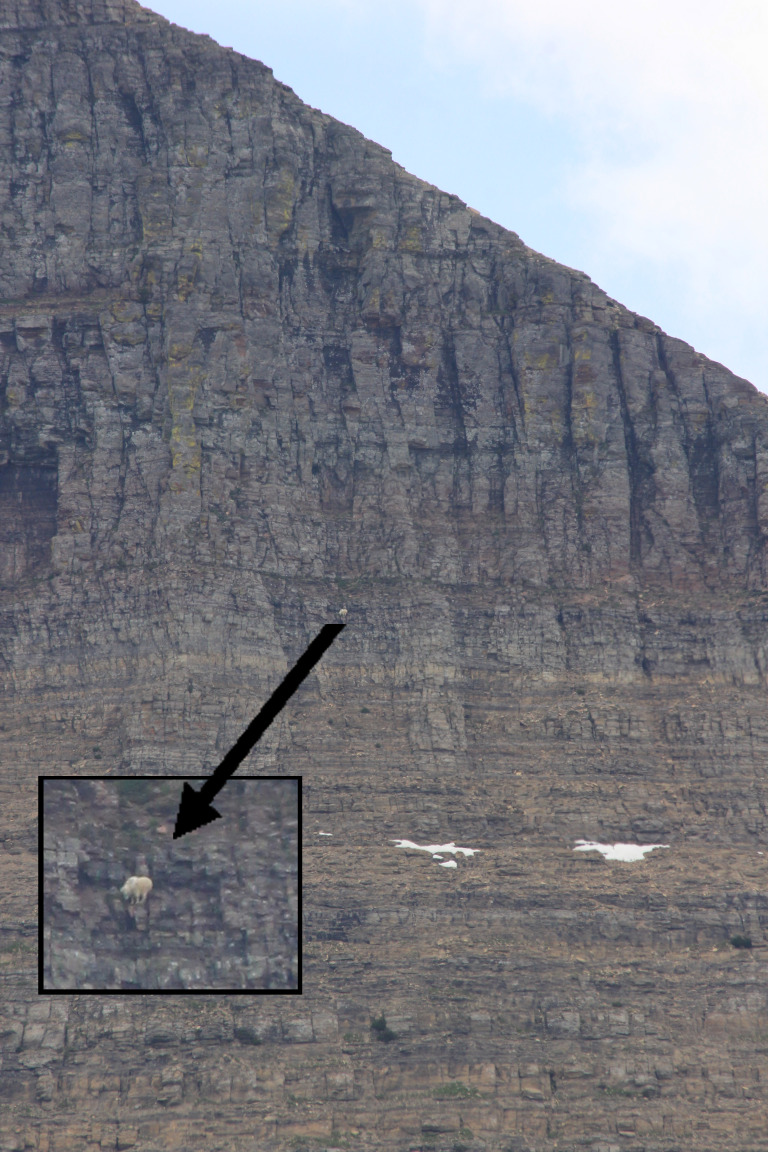
Example of mountain goat escape location (>70° slope) 12 min after being exposed to the simulated grizzly bear. Photograph in 2015 by Wesley Sarmento in Glacier National Park.

Using univariate conditional logistic regression models, we tested 22 landscape covariates to explore habitat features of escape locations used by goats after exposure to the simulated bear. The top three models explaining variation in habitat features in these trials carried 96% of the AICc weights (Table 3). Proximity to 60°slopes or greater best explained locations to which mountain goats fled (ß= −0.040, S.E. = 0.013, Z = −3.137, *P* = 0.002). On average, escape locations were 52.41m (± S.D. 92.7., Range = 0–361 m) away from  > 60°slopes, and the slope of escape locations averaged 56.51° (±14.1 S.D., Range = 22–76; Fig. 2). Goats selected for slopes between 50–80°, which was disproportionate to availability—as steepness increased, availability decreased (Fig. 3). The covariates (1) log distance to greater than 60° slopes and (2) log distance to 50° slopes and greater were the next best models. The top model had an area under the curve of 0.981, and a 0.782 cross validation estimate of accuracy.

We found 69 accounts of mountain goat-predator interactions, of which 36 resulted in predation (Table 4). Predation from terrestrial predators was unsuccessful when goats fought back (*n* = 5) or retreated to cliffs inaccessible to carnivores (*n* = 22). Goats also successfully defended kids (*n* = 6). Importantly, we did not find documented successful attacks by terrestrial predators on mountain goats when on cliffs, one of which included a 20-hour interaction between a grizzly bear and a goat in the St. Elias Range of Alaska (Table 4).

**Table 4:** Summary of mountain goat-predator interaction outcomes that have been observed or recorded in peer-reviewed literature. Columns denote the outcome of those interaction including instances where mountain goats stood their ground and successfully defended themselves (Defense). Cliff escape occurred when goat successfully fled to escape terrain. Predation occurred when the outcome of the interaction resulted in death.

**Predator**	**Cliff escape**	**Defense**	**Predation**	**Total**
Wolves^a^^,^^b^^,^^c^^,^^d^	4	1	17	22
Coyotes^e^^,^^f^	2	1		3
				
Grizzly bear^h^^,^^d^^,^^f^^,^^e^^,^^j^	15		9	24
Lynx^f^		1		1
Mountain lion^d^^,^^f^^,^^e^^,^^i^	1		8	9
Wolverine^b^^,^^f^		2		2
Golden eagle^g^^,^^f^		6	2	8
Total	22	11	36	69

**Notes.**

a Fox & Streveler (1986).

b Coté, Peracino & Simard (1997).

c Smith (1986).

d Festa-Bianchet & Côté (2008).

e Sarmento & Berger (2017).

f Chadwick (2002).

g Hamel & Côté (2009).

h Côté & Beaudoin (1997).

i Hornocker (1970).

j Berger, St. Elias, Range Alaska (2018).

## Discussion

### Antipredator behavior

While anti-predator tactics differ by species (Caro, 2005), mountain goats quickly escape to inaccessible terrain when exposed to apparent risk. Goats almost always fled immediately upon detection of our simulated bear threat, while they did not flee from our simulated ungulate control. These different responses suggest that the level of risk from our simulations were perceived by goats as we anticipated. We therefore consider goat reaction to our bear treatment a reliable representation of antipredator behavior. Neither sex nor age explained variation in the latency of response. Larger groups fled more quickly, which is likely a consequence of larger groups having quicker detection. Further, goats responded more rapidly when the bear simulation was closer, which is consistent with other studies investigating relationships between potential immediacy of threat and response intensity (Stankowich & Blumstein, 2005), but goats, with their sharp horns, are also known to fend off predators as a secondary defense (Coté, Peracino & Simard, 1997). Since interspecific aggression carries obvious risks, such behavior is likely a last resort. Other species will frequently group-up with conspecifics to reduce risk through increased predator detection and the dilution effect (Hebblewhite & Pletscher, 2002). In our experiment, mountain goats never clustered together as evidenced by the lack of change in nearest neighbor distances, which suggests the overarching importance of escape habitat relative to that by buffering by herd-mates to minimize predation risk.

Our study is unique because very few researchers have experimentally manipulated visual risk cues in wild animals on a landscape scale. Most large-scale predation risk treatments on wild ungulates have used auditory playback or scent cues (Berger, 2007; Hettena, Munoz & Blumstein, 2014). We have not found other published results on visual risk manipulation on ungulates, although such experiments have been conducted with some regularity on primates (Kemp & Kaplan, 2011). Our data align with other studies, which show that these types of visual treatments have high utility for helping us understand how species respond to predation risk. We surmise that scent or auditory risk cues would not have provoked the same reaction in mountain goats due to their life history strategy (Festa-Bianchet & Côté, 2008). Goats have evolved in mountainous terrain, areas where high wind regularly occurs, which may cause sound and scent to be a less effective sense. Indeed, goats have small ears relative to forest ungulates and we found sneaking downwind of goats was predictable and easy for close approaches. Conversely, goats have a keen sense of sight as their alpine environment lacks trees which would obscure their visual field. More than once we were visually detected by goats from over two kilometers away, which caused individuals to flee before we could properly set-up a trial. Goats fleeing before trials was a major set-back as field sites were far into the backcountry and thus required much time and resources to reach. Visual treatments of risk on wild ungulates across a landscape are logistically difficult to preform, but perhaps may be the best method for truly understanding anti-predator behavior.

### Mountain goat escape terrain

Seldom used, but critical habitat components have received little attention in ecology and conservation (Wolf et al., 1996) beyond the realm of occasionally-used seasonal migration corridors (Berger, Cain & Berger, 2006), yet such ambient features must have played prominent roles in the evolution of escape strategies or movement (Caro, 2005). Just as diurnal rodents like prairie dogs and marmots rely on burrows to escape predators (Blumstein et al., 2008), mountain goats rely on cliffs. What previously has been quite unclear is the extent to which precipices of differing degree provide refuge to alpine obligate ungulates. An impediment to understanding such phenomena has been that escape habitats may be infrequently used and, thus, their contribution to survival remains underappreciated, especially because dynamic interactions are rarely observed. It is only when an urgency for escape becomes immediate that the relative importance of topographical features become prominent, a problem that we investigated by manipulating perceived risk of mountain goats by the sudden appearance of a simulated predator. Central to this approach is the unequivocal establishment of habitat features available to, and used, by goats under heightened threat. Through our field trials and the observations of others, we ascertained that mountain goats selected for slopes of 50° up to 80° after the appearance of a simulated predator. Slopes up to 40° were almost never used, despite being highly available. Slope availability decreased with steepness, while goats selected for these steeper areas. There were no slopes above 80° and it is not clear if goats could actually use purely vertical cliffs. Our escape estimates undoubtedly are conservative since our simulations included only visual presentations, lacked the reality of predatory pursuit, and may have been insufficient to induce goats to select the steepest slopes available.

Not all escape terrain is the same, of course, nor the abilities of different predator species to use it. Grizzly bears for instance, use slopes up to 69° for denning and dig for army cutworm moths (*Euxoa auxiliaris*) in alpine scree zones (Pigeon et al., 2014), but seem very unlikely to efficiently hunt goats on such steep areas. It seems likely that slopes even steeper than 60° or 70° would enhance escape from grizzly bears and other terrestrial predators, if available. Other populations of goats, however, may not experience high predation pressure or have steep slopes available and thus there may be localized differences in goat selection of escape habitat.

At the nexus of modelling, empirical observations and reality, a concern persists about interpretation and applicability of results, whether for goats or other species reliant on discrete habitat as anti-predator refuges. Resource selection estimates for goat escape terrain would be more accurate if we consider that the slopes goats use in response to predation risk have mostly been averaged downward, by their frequent use of more gentle areas in search of food, water, and minerals. For example, the pre-stimulus data we collected could be interpreted as goat habitat in the absence of a detected predator, mostly 10–50° slopes, which is comparable to other studies which show goats use these lesser slopes the most. As is the case with steep rock cliffs, it may be difficult to appreciate their fundamental significance in the absence of observed perturbation involving predator–prey interactions or experimental provocation. Even when threatened with a simulated bear, goats sometimes did not flee directly onto cliffs—thus, even our resource selection analyses may have underestimated the steepness of escape terrain. Studies of resource selection are of clear ecological and behavioral relevance because they increase understanding of how animals use habitat (Manly et al., 2002), and their value enhanced by consideration of rarely used, but vital resources, which in our case include escape terrain.

### Applying an understanding of habitat structure to conservation

An understanding of prey-predator interactions and habitat relationships has important conservation implications (Caro, 2005). For instance, light pollution, human alterations of food, and recent extinction of large carnivores result in alterations of ecological processes that compromise habitat use and may affect survival (Clemmons & Buchholz, 1997). Our results describing how mountain goats use steep slopes when exposed to a simulated predator in field trials have direct relevance for conservation. This is particularly true not only for mountain goats but for many sensitive species threatened by human activities, especially when their specialized habitat requirements have potential to conflict with commercial activities (Christian et al., 2009), as is the case for mining, timber extraction, and skiing (White, Gregovich & Levi, 2018). Species or population restoration efforts have only infrequently focused on putting into practice criteria for suitable habitat that include rarely used refuges from predators (Poessel et al., 2011).

While restoration of wildlife has had some successes in which understanding habitat requirements has played seminal roles (Griffith et al., 1987; Wolf et al., 1996; Cheyne, 2006), there have also been failures, which are less understood. In a review of 116 animal reintroductions, (Fischer & Lindenmayer, 2000), considered habitat quality a success factor, yet few studies have examined this hypothesis. For example, introduction of caribou (*Rangifer tarandus*) into less-than-ideal habitat resulted in high mortality (Warren et al., 1996). In Australia, reintroductions of brown treecreepers (*Climacteris picumnus*) have failed in sites that had fewer predation refuges in the form on logs and trees (Bennett et al., 2013). Other unsuccessful reintroductions in Australia also did not identify suitable habitat before releases took place (Sheean, Manning & Lindenmayer, 2012). Although understudied, such sorts of case studies reaffirm that a miscalculation in the suitability of areas for reintroduction may shape prospects for success.

More specifically, in mountain goats, misestimation of appropriate escape terrain may have contributed to some of the 27 unsuccessful introductions of 70 total attempts (Guenzel, 1980; Harris & Steele, 2014). These presumed introduction failures likely resulted from small initial population size and focused less on considerations of habitat quality or escape terrain. Nonetheless, a likely example in which escape terrain did play a role comes from an unsuccessful goat introduction in the ∼76 km^2^ National Bison Range (NBR), Montana, USA (McCullough, 1980). Mountain goats inhabited NBR for more than 30 years and attained a population high of 74 individuals in 1995 (NBR unpublished data). The following year, goat numbers dropped to 24, which coincided with mountain lion re-colonization. Goats were last detected in 2005. Because the entire reserve was fenced, dispersal cannot account for the population decline. A lack of escape terrain in the presence of mountain lions is the likely explanation because less than 1% of the area at NBR is escape terrain (slopes >60°), and the total amount of area with slopes greater than 50° is 0.30 hectares. No slopes exceed 56°. Conversely, at our study site with >60° slopes degrees widely available, goats have always persisted despite mountain lions and the full suite of native predators.

Resource selection studies have provided important insights, but the critical assumption in these analyses—that the time an animal spends with a resource relative to availability is proportional to importance—has received little attention (Wolf et al., 1996; Beyer et al., 2010). Understanding how habitat features are defined and the associated assumptions and limitations is an important consideration for the optimization of conservation outcomes. Here, we show that rare interactions with predators may bias what we calculate as important habitat for mountain goats. Escape terrain may be used infrequently, but these places might be essential to the survival of mountain goats in locations where predation risk is high. Our experimental approach may be one method to help identify habitat selection under rarely occurring circumstances. Future resource selection studies could be enhanced by considering rarely used, but critical habitat features that might not show up in typical analyses of collar data or field observations.

## Conclusions

Habitat critical for safety from predators is a well-tested evolutionary phenomenon by which prey frequently avoid attack from predators. Selection operates on successes. Habitat is always a requisite part of success, yet sometimes the appropriate identification of habitat remains challenging. Predation risk simulation experiments appear a prudent technique to enhance understanding of critical habitats. Resource selection studies may fail to identify safe habitat because the importance of landscape features is based on proportional use, when direct risk to an individual occurs relatively rarely and therefore the use of security areas occurs infrequently.

Misidentification of security habitat has economic, ecological and conservation implications such as (1) failed (re)introductions into habitat that cannot support a prey species in the presence of predators, (2) over- or under-harvest of hunted species from miscalculating safe habitat, (3) unnecessary curtailment of commercial activities (such as ski resorts, timber harvest, or mining) resulting from overestimating available refuge habitat, and (4) lack of adequate habitat protection by underestimating refuge habitat needs or availability. Overharvest, for example, could occur if wildlife managers over-estimate the amount of safe habitat and thus would predict harvest success to be lower than what it would actually be, and vice versa. For mountain goats that experience high predator densities and have steep slopes available, such as in Glacier National Park, we improved the precision on what has previously been a wide range of slope values for defining escape habitat; slopes greater than 60° best explained the sites to which goats fled when exposed to immediate apparent risk. We believe our estimates of escape terrain were slightly averaged downward because goats staged next to cliffs as we did not pursue individuals with our simulated bear. It is, therefore, tenable that true goat escape terrain, when available, would exceed 70°. Future work might profitably analyze previous goat introduction sites to determine if lack of escape terrain was a contributing factor of the outcome. Additionally, there is much to be learned about how the type, amount, and distribution of escape terrain relative to other resources influence mountain goat populations, which is especially important in areas where native goat populations are declining. Finally, there are other key resources, such as salt licks, that are undervalued, but vitally important to the habitat selection and survival of various species beyond mountain goats.

##  Supplemental Information

10.7717/peerj.9296/supp-1 Table S1 Top model results for mountain goat latency to respons Coefficient estimates for top linear model explaining mountain goat latency to response after exposure to bear imitation experiment (*N* = 37). Group size of mountain goats included all individuals present during the experiments that took place in Glacier National Park from 2014–15.

10.7717/peerj.9296/supp-2 Table S2 Model results for mountain goat latency to response explained by distance to experiment Coefficient estimates for competing linear model explaining mountain goat latency to response after exposure to bear imitation experiment (*N* = 37). Distance to imitation was the distance (meters) between focal individual and the experiment estimated using a rangefinder. Thirty-seven experiments took place in Glacier National Park from 2014–15.

10.7717/peerj.9296/supp-3 Supplemental Information 1 Raw data on mountain goat response to experimental limitations Sample sizes are: baseline = 67, ungulate = 30, bear = 37. This study was conducted in Glacier National Park, Montana, USA during June, July, and August of 2014–15.

## Additional Information and Declarations

### Field Study Permissions

The following information was supplied relating to field study approvals (i.e., approving body and any reference numbers): Glacier National Park provided a permit for this work (GLAC-2013-SCI-007).

### Data Availability

The following information was supplied regarding data availability: Data is available in the Supplementary Files.
