# Supplementary material for: Conservation implications of using an imitation carnivore to assess rarely used refuges as critical habitat features in an alpine ungulate
Source: PeerJ. 2020 Jun 12;8:e9296. doi: 10.7717/peerj.9296 (PMC7295023; doi:10.7717/peerj.9296)
Supplement: Table S1 — Coefficient estimates for top linear model explaining mountain goat latency to response after exposure to bear imitation experiment (N = 37). Group size of mountain goats included all individuals present during the experiments that took place in Glacier National Park from 2014–15. [file peerj-08-9296-s001.docx]

**Supporting Information Table 1.** **Top model results for mountain goat latency to response.** Coefficient estimates for top linear model explaining mountain goat latency to response after exposure to bear simulation experiment (N=37). Group size of mountain goats included all individuals present during the experiments that took place in Glacier National Park from 2014-15.

|  | **ß** | **S.E.** | **Z** | **P** |
| --- | --- | --- | --- | --- |
| Intercept | 0.820 | 0.722 | 1.136 | 0.256 |
| Group size | 0.144 | 0.155 | 0.928 | 0.353 |
