# Supplementary material for: Conservation implications of using an imitation carnivore to assess rarely used refuges as critical habitat features in an alpine ungulate
Source: PeerJ. 2020 Jun 12;8:e9296. doi: 10.7717/peerj.9296 (PMC7295023; doi:10.7717/peerj.9296)
Supplement: Table S2 — Coefficient estimates for competing linear model explaining mountain goat latency to response after exposure to bear imitation experiment (N = 37). Distance to imitation was the distance (meters) between focal individual and the experiment estimated using a rangefinder. Thirty-seven experiments took place in Glacier National Park from 2014–15. [file peerj-08-9296-s002.docx]

**Supporting Information Table 2.** **Model results for mountain goat latency to response explained by distance to treatment**. Coefficient estimates for competing linear model explaining mountain goat latency to response after exposure to bear simulation (N=37). Distance to simulation was the distance (meters) between focal individual and the treatment estimated using a rangefinder. Thirty-seven experiments took place in Glacier National Park from 2014-15.

|  | **ß** | **S.E.** | **Z** | **P** |
| --- | --- | --- | --- | --- |
| Intercept | 3.049 | 0.917 | 3.324 | 0.001 |
| Distance to simulation | -0.008 | 0.003 | -2.342 | 0.019 |
